# Effect of *Eucalyptus globulus* oil and *Ricinus communis* methanolic extract as potential natural molluscicides on the reproductive biology and some antioxidant enzymes of the land snail, *Theba pisana*

**DOI:** 10.1016/j.heliyon.2022.e12405

**Published:** 2022-12-20

**Authors:** Mahmoud M.A. Desouky, Mahmoud S. Abd El-Atti, Ali A. Elsheakh, Wesam S. Elgohary

**Affiliations:** a Zoology Department, Faculty of Science, Zagazig University, Zagazig, 44519, Egypt; b Plant Protection Research Institute, Agriculture Research Center, Dokki, Giza, Egypt

**Keywords:** Theba pisana, Eucalyptus globulus oil, *Ricinus communis*, Antioxidants, Hermaphrodite gland, Histopathology

## Abstract

This study was conducted to investigate the effects of *Eucalyptus globulus* oil and methanolic extract of *Ricinus communis* seeds on the reproductive biology of the land snail *Theba pisana*. For this purpose, the snails were exposed to different concentrations of these plant extracts for six weeks. Rates of oviposition, hatching percentages, reproductive hormones and the histological structures of the hermaphrodite gland were estimated. Antioxidant enzymes were also estimated. The obtained results revealed that all tested concentrations of both tested natural products exerted hazardous effects on exposed snails. The mean egg number/snail treated with 2% *E. globulus* and *R. communis* were significantly decreased to 22 and 14 eggs respectively compared to 79 eggs for control. The hatching rates were dramatically decreased with increasing concentrations of both products. 2% of *R. communis* extract caused highly significant decrease in the activities of CAT, SOD, GST and MDA antioxidant enzymes while the same concentration of *Eucalyptus* oil resulted in elevations of CAT and SOD activities and significantly decreased GST and MDA activities. Levels of reproductive hormones were greatly disrupted and the histological structures of hermaphrodite acini as well as various developmental stages of spermatogenesis and oogenesis of treated snails were strictly spoiled.

## 1 Introduction

Terrestrial gastropods are very hurtful pests for crops, vegetables, fruit, ornamental plants, and ecosystem. (Srivastava, 1992; Zala et al., 2018). They cause contaminations to agricultural products by their feces or slime, resulting in alterations in their qualities and financial loss (Carlsson et al., 2004; Ali, 2017; Kumar, 2020). Land snails act also as intermediate hosts for digenian trematodes such as sheep lung worms, the lancet liver flukes and cattle lung worms (Godan, 1983). The white garden snail *Theba pisana* is the most widely spread land snail in the Mediterranean region (Routray and Dey, 2016). High population densities of *T. pisana* were reported to cause considerable damage to agricultural and horticultural plants at Sharkia governorate, Egypt (Ismail et al., 2015). It also causes annoyance to farmers due to its small size, fast climbing behaviour, high survival rates, fast dispersal and explosive reproductive rates (Deisler et al., 2015). In addition *T. pisana* snails has the ability to respond well and avoid certain chemical cues (Lefcort et al., 2006).

Chemical molluscicides used for long times in controlling land snails have many disadvantages such as disruption of natural biological control systems, undesirable effects on non-target organisms and development of pests’ resistance to synthetic insecticides (Ismail et al., 2015; Abd El-Atti et al., 2019). About 25 million of agricultural workers were poisoned by pesticides in developing countries every year (Farag, 2017). Nowadays, ecofriendly botanical pesticides have constituted alternative methods for controlling agricultural pests (Edyta et al., 2018).

Several local but exotic plant species in Egypt are screened and demonstrated potential molluscicidal activities against different species of snails (Abd El-Atti et al., 2019). Exotic *Eucalyptus globulus* (Blue gum trees) native to Australia and endogenous *Ricinus communis* (Castor) are cosmopolitan plants growing wildly on stream banks at Sharkia Governorate and their products can be obtained easily by farmers for controlling extensive populations of *T. pisana* snails. Essential oils have been reported as available and important sources of biopesticides due to their richness of bioactive compounds which are biodegraded into nontoxic products (Lahlou, 2004). *Eucalyptus globulus* trees (Family Myrtaceae), are rich in oil glands and are excellent sources of commercially important oil having fungicidal, antibacterial and antiseptic characters (Brooker and Kleinig, 2006). Eucalyptus oil has been placed under GRAS (Generally Regarded as Safe) category by Food and Drug Authority of USA and classified as non-toxic (USEPA, 1993). Essential oil of *Eucalyptus* is used as insect repellent and as a pesticidal agent (Barton, 2000; Abdel Halim and Morsy, 2005; Lucia et al., 2007; Mahfuz and Khalequzzaman, 2007) as well as ticks repellent (Lefcort et al., 2020). Many studies revealed that *Eucalyptus* oil affects the fertility of some insects (Campolo et al., 2018; Hategekimana and Erler, 2020) and was reported to be potential sources for biocidal compounds against the fluke *Schistosoma mansoni* and its snail host *Biomphalaria alexandrina* (Al-Sayed et al., 2014). On the other hand, Castor (*Ricinus communis*) (family Euphorbiaceae) seeds have antihelminthic, emollient, laxative, cathartic and purgative properties (Chiej, 1984). *R. communis* methanol extract is used in treatment of some diseases like diabetes mellitus (Parker et al., 2016). *Ricinus* beans extract have been reported to be used as contraceptive agent in India and Korea (Farnsworth et al., 1975; Woc et al., 1981). Moreover, the methanolic extract of *Ricinus* seeds inhibited the implantation of embryos and induced abortion in guinea pigs and women (Makonnen et al., 1999; Isichei et al., 2000; Das et al., 2000). *R. communis* seed extract has a negative impact on the reproductive functions of male rats and mediated via gonadal disturbance in testosterone secretion (Raji et al., 2006). Furthermore, the castor bean seeds contain compounds with anti-tryptic activity and reduced the oviposition period of the armyworm *Spodoptera frugiperda* (Ramos et al., 2013). Moreover, Jehan *et al.* (1973) and Sani and Sule (2007) stated that steroids and alkaloids in methanol extract of *Ricinus* seeds have the same action of combined estrogen and progesterone, in addition to glycoprotein ricin a poisoning material interfered with enzymes reactions. Regarding Eucalyptus, Hategekimana and Erler (2020) reported the antifertility effect of its major component (1, 8-cineole) on some insects.

Oxidative stress resulting from the enhancement of ROS (Reactive Oxygen Species) and disturbance of antioxidant efficiency is recognized as a vital general toxicity mechanism of many xenobiotic, including pesticides (Regoli et al., 2002, 2006). It is known that oxidative stress often preludes the onset of long term effects such as degenerative processes, impairments of immune response and reproduction, premature aging and lower survival rate (Banerjee et al., 1999). Therefore, measurement of antioxidant enzymes is useful in determination of oxidative stress induced by these plant extracts.

In view of the aforementioned, the present study was designed to evaluate the effect of these two plant extracts on different aspects of reproductive biology of *T. pisana* snails as well as some antioxidant enzymes that represent the first defense line against oxidative stress. This study supposes that the biology, antioxidant enzymes and reproductive hormones of these snails will greatly affected by these two plants derivatives. This may be of great value in biological control to reduce the population density of *Theba pisana* snail which represents a dangerous pest in Sharkia Governorate, Egypt.

## 2 Materials and methods

### 2.1 Collection and acclimation of snails

Adult *T. pisana* snails (15–18 mm in diameter) were collected from El-Mohammadia village, Sharkia Governorate, Egypt, (30°30′55″N, 31°20′46″E) during October 2021. The collected snails were transferred alive to the Pest Physiology Lab., Plant Protection Research Institute, Zagazig, Egypt. They were kept in glass jars (40 × 30 × 30 cm) with 10 cm moist soil at bottom and covered with muslin cloth. They were maintained at 20 ± 2 °C and relative humidity 80–90 % and fed daily on fresh organic lettuce leaves for two weeks.

### 2.2 Bait formulation

Pure *Eucalyptus globulus* oils and *Ricinus communis* seeds extract were purchased from the International Company (Cairo-Egypt). Baits were prepared by adding (94.5, 94 and 93) gram wheat bran to 5 g sugarcane syrup mixed with 0.5, 1 and 2 g of either methanolic extract of *Ricinus* seeds or *Eucalyptus* oil for preparing the examined concentrations of (0.5, 1 and 2%, respectively).

### 2.3 Experimental design

Snails were divided into seven groups: Control and six treated groups (exposed to either 0.5, 1 or 2% of both tested plant extract). Each group comprised 5 replicas (n = 5); each replica is a plastic box (750 g capacity) contains 500 g of moist soil and small plate with 10 g of bait. Four snails were transferred from the glass jar after adaptation and added to each replicate and left to feed on baits for 6 weeks. These boxes or replicates were placed under laboratory conditions 20 ± 2 °C and relative humidity 80–90 %. Egg numbers were counted weekly during the period from 15 November to the end of December. Egg masses were removed weekly from soil and put in Petri dish then counted and left to be hatched. Juveniles were counted and incubation periods as well as egg hatching rates were recorded over the exposure period (six weeks).

### 2.4 Assay of antioxidant enzymes

After six weeks of exposure, digestive glands of *T. pisana* (n = 3) were dissected out from both control and treated snails and homogenized in distilled water using a Teflon homogenizer. The homogenates were centrifuged at 4000 rpm for 10 min at 5 °C in a refrigerated centrifuge. Deposits were discarded and supernatants were kept in a deep freezer until use. The activities of catalase (CAT), superoxide dismutase (SOD), glutathione-S- transferase (GST) and lipid peroxidation (Malondialdehyde, MDA) were analyzed spectrophotometrically according to the corresponding assay kit protocol (Bio Vision-Milpitas, CA, USA).

### 2.5 Assay of reproductive hormones

At the end of exposure period, soft tissues of both control and treated snails (n = 3) were homogenized in Phosphate buffered saline, centrifuged at 8000 rpm at 5 °C and the supernatants were stored at −80 °C. Levels of Luteinizing hormone (LH), Follicle stimulating hormone (FSH), Thyroid stimulating hormone (TSH), Prolactin (PRL), Estradiol (E2) and Testosterone (T) were measured using kits, two immunological step sandwich type assay (Immunotech version; Beckman Coulter, Marseille, France).

### 2.6 Histological investigations

Pieces of the hermaphrodite glands were chosen randomly one from each replicate from both control and treated snails-about 3 samples/3 replicas-were dissected out and fixed in alcoholic Bouin's solution. Specimens were dehydrated in an ascending series of ethanol, cleared in Xylene for 20 min and embedded in paraffin wax. Sections (5 μ m thick) were cut, mounted, and stained with Hematoxylin and Eosin.

### 2.7 Statistical analysis

Data were analyzed using IBM SPSS (version 20, Armonk, NY, USA). Comparisons of the effects of different concentrations on egg numbers were performed using Two-way analysis of variance (ANOVA) One-way analysis of variance (ANOVA)was used to compare their effect on the biological aspects and the biochemical parameters (Enzymes and Hormones). Tukey's HSD Test was used to determine statistically significant deference between values. Data was considered significant at p ≤ 0.05. Variables were expressed as Mean ± S.E.

## 3 Results

### 3.1 Rates of oviposition/week

**Figure 1:**
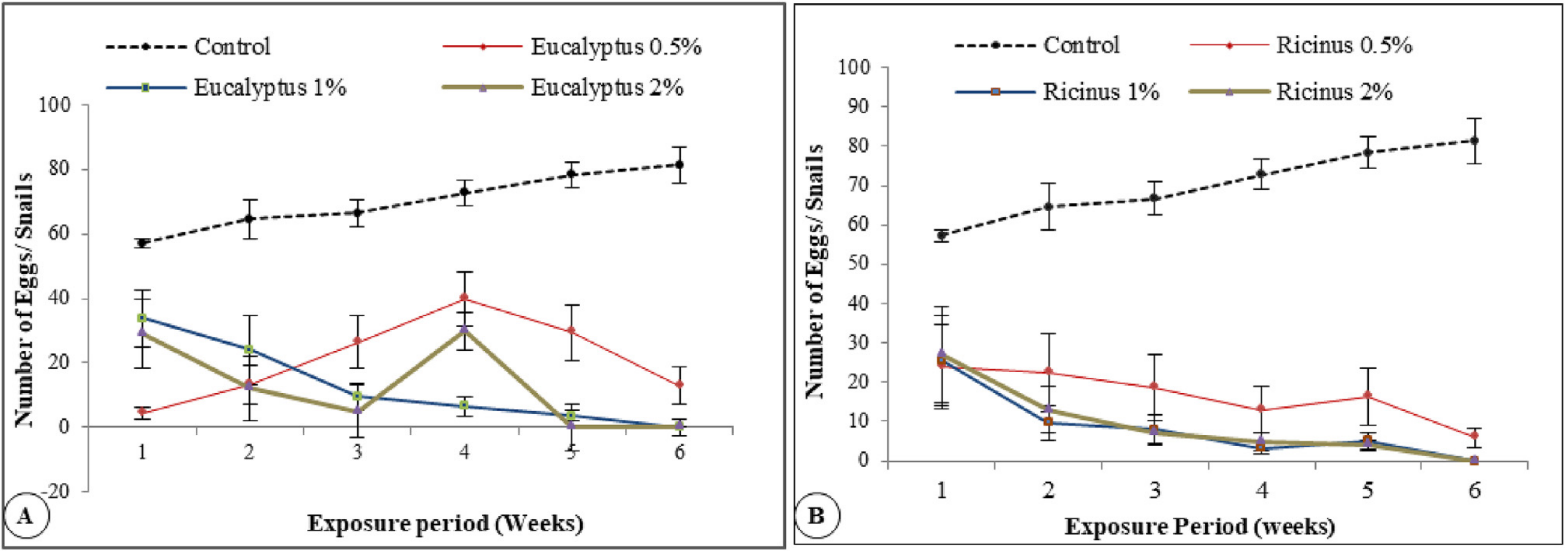
Mean egg numbers/week (M± S.E) of adult *Theba pisana* snails exposed to different concentrations of (A) *Eucalyptus* oil and (B) *Ricinus* seed extract for 6 weeks. Two-Way ANOVA was used to analyze this experiment. Values were significant at P ≤ 0.05. (A) Eucalyptus Treatments: F = 23.342418 P = 0 .0000 ∗∗∗, Exposure time (weeks): F = : 0.5738625, P = 0 .7502 ns Interaction treatment ∗ weeks: F = 1.0089902 P = 0.4576 ns. (B) Ricinus Treatments: F = 27.053618 P = 0 .0000 ∗∗∗, Exposure time (weeks): F = 0.2995321, P = 0.9356 ns. Interaction treatment ∗ weeks: F = 0.4487268 P = 0.9721 ns.

Mean number of eggs of treated snails were decreased significantly with increasing concentrations (P < 0.05) of *Eucalyptus* oil and *Ricinus* seeds extract compared to control. However statistics revealed that time of exposure (weeks) was non-significant in its effect on number of eggs. 0.5% *Eucalyptus* oil caused highly significant decrease (P < 0.05) (4 eggs/snails) at the first week of exposure compared to control (57 eggs/snails). Egg numbers were gradually increased up to (39 eggs/snails) at the 4th week then deceased again (13 eggs/snails) at the 6th week of treatment. The highest concentration (2%) of *Eucalyptus* oil caused gradual decrease in egg numbers until the 3rd week but suddenly increased to (30 eggs/snails) in the 4th one then completely stopped (0 egg/snails) at the 5th week of exposure (Figure 1A).

On the other hand, *Ricinus* extract (0.5%) severely decreased the number of eggs up to (5 egg/snails) at the 6th week of exposure while the highest concentration of *Ricinus* (2%) caused complete ceasing of egg laying at the 6th week of treatment (Figure 1B).

### 3.2 Numbers of egg masses and hatching rates of treated T. pisana

Numbers of egg masses/snail of *T. pisana* were decreased after treatment with different concentrations of *Eucalyptus* oil and *Ricinus* seeds extract compared to control. The egg masses number/snail were significantly decreased (P < 0.05) to22.4 and 14.45, respectively after exposure to 2% *Eucalyptus* oil and *Ricinus* extract compared to control (79.35). The incubation period was decreased significantly with increasing concentrations of the tested extracts. The highest concentration (2%) caused significant decrease (P < 0.05) of hatching rates by (51.89%) and (31.26%) for *Eucalyptus* and *Ricinus* extracts respectively*,* compared to control (96.24%) after six weeks of treatment (Table 1).

**Table 1:** Effects of treatment with different concentrations of *Eucalyptus* oil and *Ricinus* seeds extract on egg laying capacities, incubation period and hatching rates of *Theba pisana* snails after 6 weeks of exposure.

Treatment	Egg mass./Snail	Egg no./Mass.	Egg no./Snail	Incubation period (days)	Hatching Rate %
Control	1.25^a^ ± 0.05	63.48^a^ ± 1.47	79.35^a^ ± 3.65	14.32^a^ ± 0.05	96.24^a^ ± 0.34
0.5% *Eucalyptus*	0.45^b^ ± 0.04	61.86^a^ ± 4.16	28.3^b^ ± 3.08	13.95^a^ ± 0.12	73.51^ab^ ± 2.67
1% *Eucalyptus*	0.4^b^ ± 0.03	61.05^a^ ± 6.59	22.13^b^ ± 1.29	11.8^c^ ± 0.11	67.48^b^ ± 0.35
2% *Eucalyptus*	0.55^b^ ± 0.04	38.52^a^ ± 1.78	22.4^b^ ± 2.26	9.66^d^ ± 0.22	51.89^bc^±2.24
0.5 % *Ricinus*	0.35^b^ ± 0.03	58.2^a^ ± 3.78	20.35^b^ ± 1.84	12.2^bc^±0.15	75.6^ab^ ± 2.22
1% *Ricinus*	0.4^b^ ± 0.03	45.7^a^ ± 2.34	18.25^b^ ± 1.7	13.5^ab^ ± 0.14	55.21^bc^±4.82
2% *Ricinus*	0.35^b^ ± 0.03	40.4^a^ ± 5.52	14.45^b^ ± 2.41	12.3^bc^±0.17	31.26^c^ ± 1.07
LSD 0.05	**0.232**	**26.42**	**15.81**	**0.95**	**15.80**
P	**.0000 ∗∗∗**	**0.2476 ns**	**.0000 ∗∗∗**	**.0000 ∗∗∗**	**.0000 ∗∗∗**

Data are presented as mean ± S.E. Mean values with different alphabetical superscripts are statistically significant at P < 0.05. statistical analysis was carried out by one way ANOVA.

### 3.3 Antioxidant enzymes

**Figure 2:**
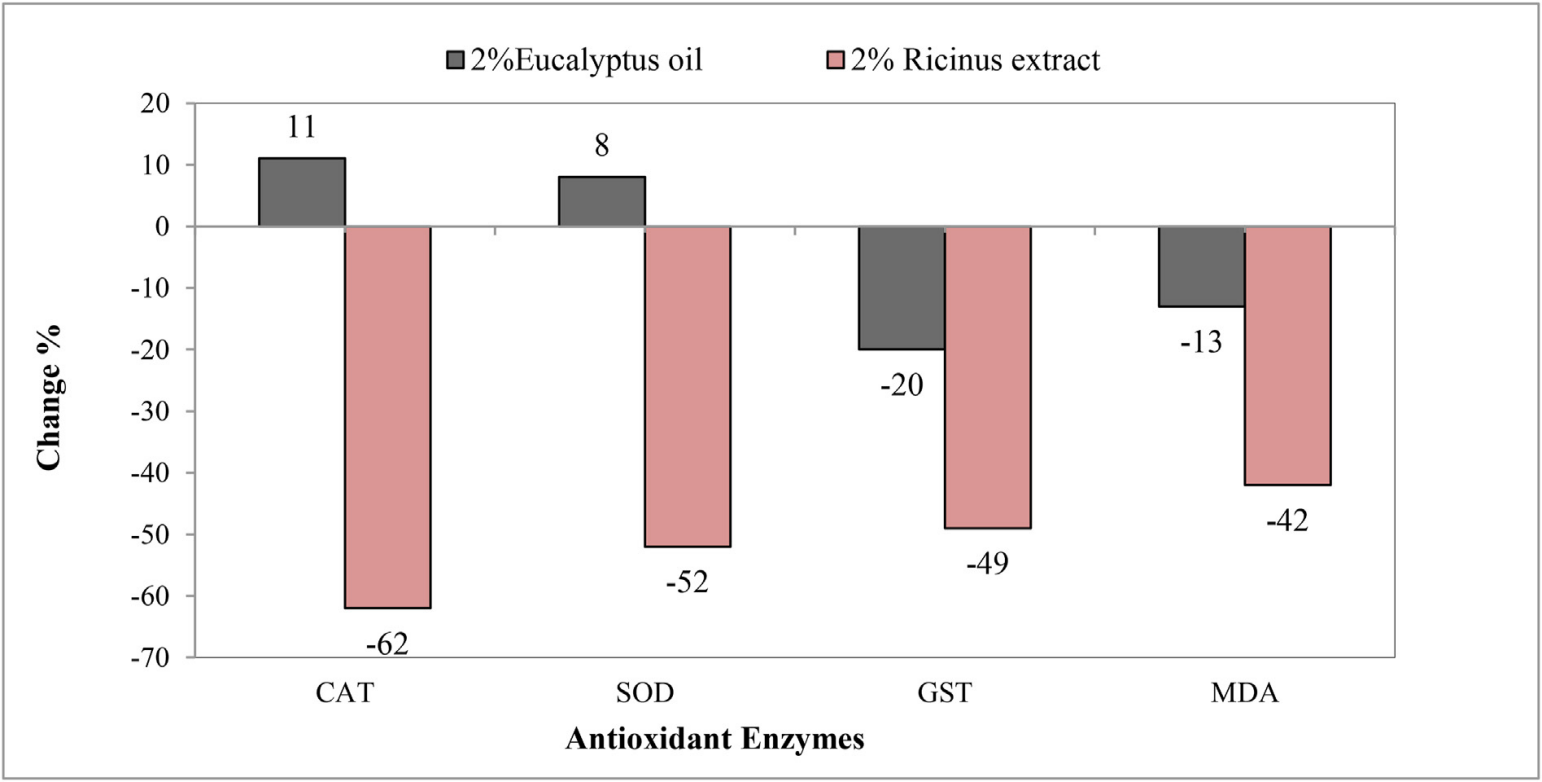
Changes (%) of the activities of some antioxidant enzymes in *T. pisana* treated with either 2% *Eucalyptus* oil or *Ricinus* seeds extract for 6 weeks.

**Figure 3:**
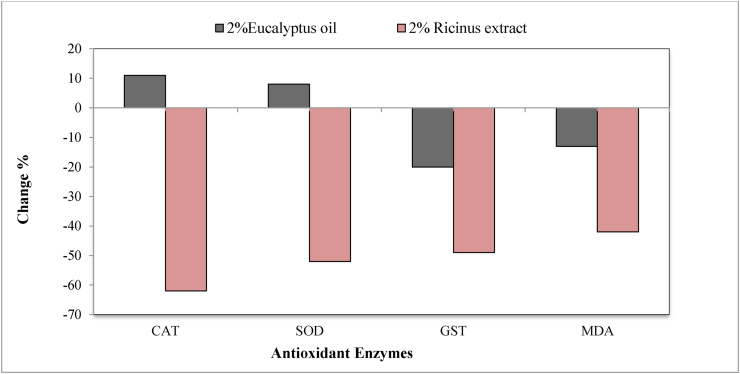
Changes (%) of the activities of some antioxidant enzymes in *T. pisana* treated with either 2% *Eucalyptus* oil or *Ricinus* seeds extract for 6 weeks.

Specific activities of catalase (CAT), superoxide dismutase (SOD), glutathione-S- transferase (GST) and lipid peroxidase (MDA) of *T. pisana* were significantly decreased (P < 0.05)after treatment with 2% *Ricinus* extract for six weeks. (Table 2). Percentages of decrease were (62, 52, 49 and 42%), respectively compared to control. However, Snails exposed to 2% *Eucalyptus* oil showed significant increase (P < 0.05) in activities of (CAT) and (SOD) (Table 2) by percentages (11 and 8%) while significantly decreased activities (P < 0.05) of both glutathione-S- transferase and malondialdehyde with decrease percentages (20 and 13%) at the end of exposure period (Figures 2,3).

**Table 2:** Specific activities of some antioxidant enzymes in *T. pisana* treated with either 2% *Eucalyptus* oil or *Ricinus* seeds extract for 6 weeks.

Treatment	Conc.%	Egg mass./Snail	Egg no./Mass.	Egg no./Snail	Incubation period (days)	Hatching Rate %
**Control**		1.25^a^ ± 0.05	63.48^a^ ± 1.47	79.35^a^ ± 3.65	14.32^a^ ± 0.05	96.24^a^ ± 0.34
***Eucalyptus***	**0.5**	0.45^b^ ± 0.04	61.86^a^ ± 4.16	28.3^b^ ± 3.08	13.95^a^ ± 0.12	73.51^ab^ ± 2.67
	**1**	0.4^b^ ± 0.03	61.05^a^ ± 6.59	22.13^b^ ± 1.29	11.8^c^ ± 0.11	67.48^b^ ± 0.35
	**2**	0.55^b^ ± 0.04	38.52^a^ ± 1.78	22.4^b^ ± 2.26	9.66^d^ ± 0.22	51.89^bc^ ± 2.24
***Ricinus***	**0.5**	0.35^b^ ± 0.03	58.2^a^ ± 3.78	20.35^b^ ± 1.84	12.2^bc^ ± 0.15	75.6^ab^ ± 2.22
	**1**	0.4^b^ ± 0.03	45.7^a^ ± 2.34	18.25^b^ ± 1.7	13.5^ab^ ± 0.14	55.21^bc^ ± 4.82
	**2**	0.35^b^ ± 0.03	40.4^a^ ± 5.52	14.45^b^ ± 2.41	12.3^bc^ ± 0.17	31.26^c^ ± 1.07
**LSD 0.05**		**0.232**	**26.42**	**15.81**	**0.95**	**15.80**
**P value**		**0.009∗∗∗**	**0.126 ns**	**0.018∗∗∗**	**0.000∗∗∗**	**0.000∗∗∗**

Data are presented as mean ± S.E. Mean values with different alphabetical superscripts are statistically significant at P < 0.05. statistical analysis was done by One-Way ANOVA.

### 3.4 Reproductive hormones

**Figure 4:**
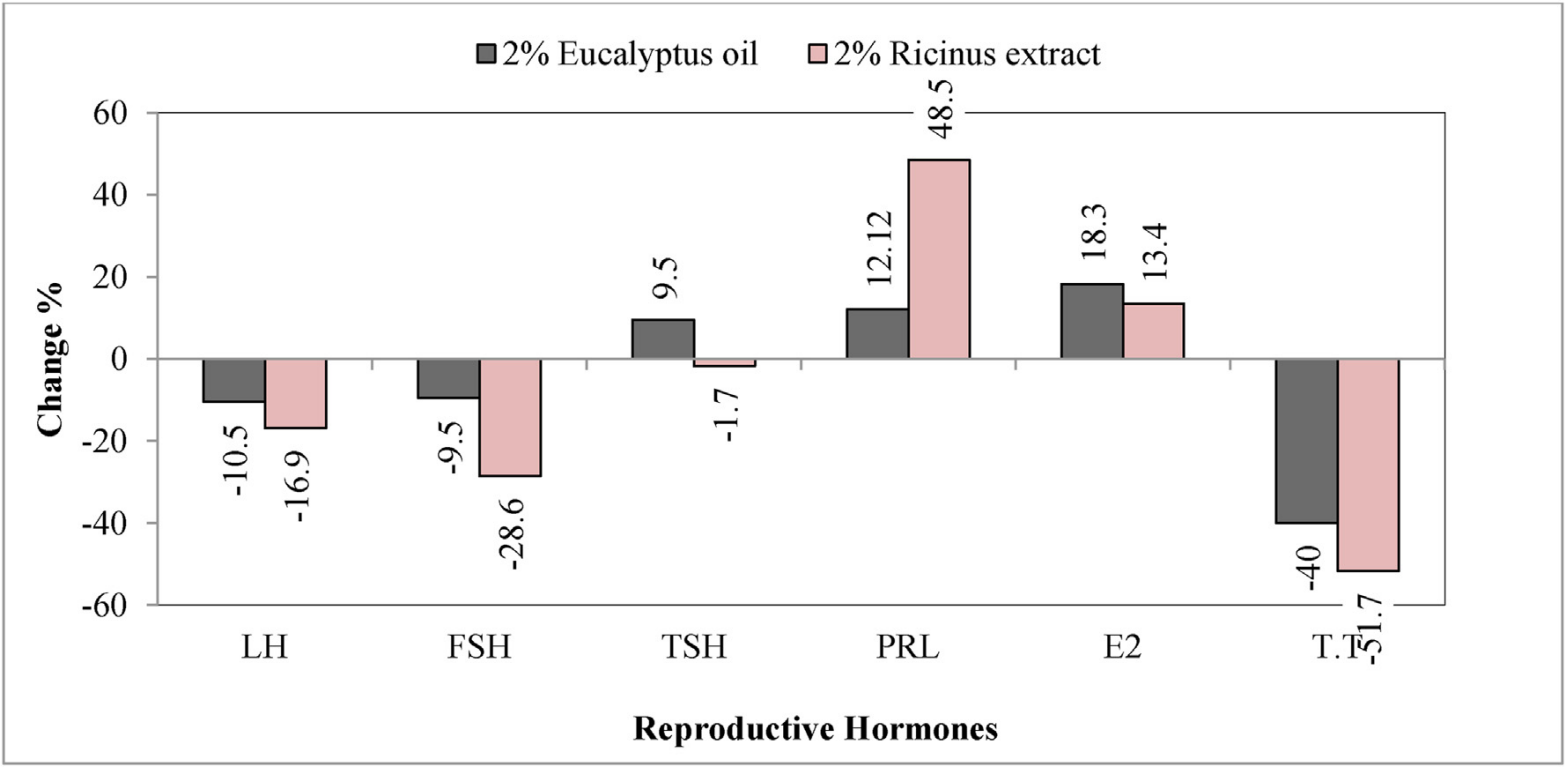
Changes (%) of some reproductive hormones levels in *T. pisana* treated with either 2% *Eucalyptus* oil or *Ricinus* seeds extract for 6 weeks.

Hormonal analysis of *T. pisana* sails treated with 2% *Eucalyptus* oil and *Ricinus* extract showed that the two natural products induced disturbances in reproductive hormones (Table 3). Ricinus extract significantly increased the levels of luteinizing hormone (LH), follicle stimulating hormone (FSH) and testosterone (P < 0.05) by percentage of decrease reached (19.6, 28.6 and 51.7%), respectively while levels of prolactin and estradiol were significantly increased by percentages (48.5 and 13.4%) respectively at the end of exposure period. Level of TSH was insignificantly decreased (P > 0.05). Eucalyptus oil caused significant decrease (P < 0.05) in LH and T.T with percentages of decrease (10.5 and 40%) respectively and significant increase in Estradiol by percentage of 18.3%. Regarding to TSH and PRL, these were insignificantly increased (P > 0.05) moreover, FSH was also insignificantly decreased. It is noticed that, *Ricinus* extract was more effective than *Eucalyptus* oil in its effect on hormone levels (Figure 4).

**Table 3:** Mean values of reproductive hormones levels in *T. pisana* treated with either 2% Eucalyptus oil or *Ricinus* seeds extract for 6 weeks.

	CAT	SOD	GST	MDA
**Control**	4.44^b^ ± 0.062	130^b^ ± 0.67	1.32^a^ ± 0.013	1.18^a^ ± 0.023
**2% *Eucalyptus***	4.93^a^ ± 0.01	140.3^a^ ± 0.31	1.06^b^ ± 0.024	1.03^b^ ± 0.012
**2% *Ricinus***	1.71^c^ ± 0.007	63^c^ ± 0.33	0.86^c^ ± 0.002	0.69^c^ ± 0.007
**LSD** **0.05**	0.226	3.12	0.144	0.108
**P**	0.0000 ∗∗∗	0.0000 ∗∗∗	0.0007 ∗∗∗	0.0001 ∗∗∗

	LH	FSH	TSH	PRL	E2	T.T
**Control**	0.313^a^ ± 0.0003	0.21^a^ ± 0.001	0.116^a^ ± 0.0003	0.033^b^ ± 0.0003	9.72^b^ ± 0.04	0.0145^a^ ± 0.0002
**2% *Eucalyptus*.**	0.29^b^ ± 0.001	0.19^a^ ± 0.003	0.127^a^ ± 0.001	0.037^b^ ± 0.0003	11.5^a^ ± 0.07	0.0087^b^ ± 0.0001
**2% *Ricinus***	0.26^c^ ± 0.0005	0.15^b^ ± 0.0029	0.114^a^ ± 0.003	0.049^a^ ± 0.001	11.02^a^ ± 0.006	0.007^c^ ± 0.0001
**LSD** **0.05**	0.0069	0.022	0.013	0.0048	0.428	8.98
**P**	0.0000 ∗∗∗	0.0016 ∗∗	0.0928 ns	0.0004 ∗∗∗	0.0001 ∗∗∗	0.0000 ∗∗∗

Data are presented as mean ± S.E. Mean values with different alphabetical superscripts are statistically significant at P < 0.05. Statistical analysis was done by One-Way ANOVA.

### 3.5 Histopathology of the hermaphrodite gland

#### 3.5.1 Normal hermaphrodite gland of *T. pisana*

**Plate 1:**
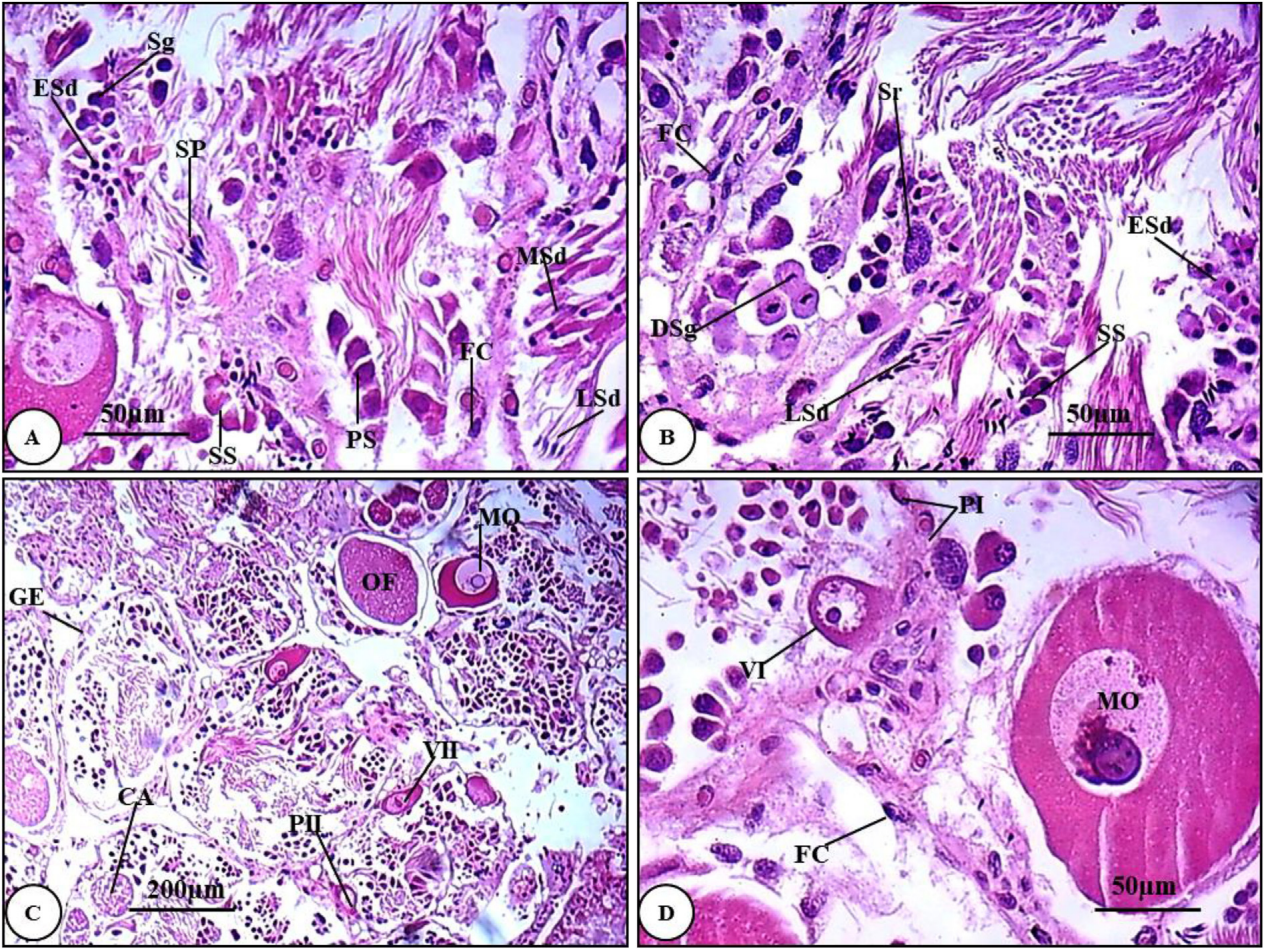
T.S. of the hermaphrodite gland of untreated *Theba pisana* stained H&E showing different stages of spermatogenesis and oogenesis. (A & B) show different stages of spermatogenesis (X = 400). (C) shows general view of the gland with different stages of oogenesis (X = 100). (D) T.S showing more detailed view of an acinus with some stages of oogenesis. *CA: Corpus albicans; DSg: Divided Spermatogonia; ESd: Early stage spermatid; FC: Follicular Cell; GE: Germinal epithelium; LSd: Late stage spermatid; Mo: Mature ovum; MSd: Middle stage of spermatid; OF: Ovulated follicle; PII: Previtellogenic Oocyte II; PSC: Primary spermatocytes; Sg: Spermatogonia; SP: Sr: Sertoli cell; Spermatozoa; SS: Secondary spermatocytes; VII: Vitellogenic oocyteII.*

The hermaphrodite gland of *T. pisana* is embedded in the digestive gland. It consists of several acini lined with squamous follicular epithelium having oval flattened nuclei with irregular chromatin patches. Sertoli cells are inserted between follicular cells and are characterized by irregular and indefinite sizes with ovoid nuclei comprising dense euchromatin (Plate 1B). Stages of the spermatogenesis are easily distinguishable. Spermatogonia are small semi rounded cells with highly basophilic nuclei. These cells undergo successive mitotic divisions near the inner acinal wall to form clusters around Sertoli cell (Plate 1A & B). Primary spermatocytes are slightly enlarged, somewhat rounded to pear shaped with a large nuclei (Plate 1A). Secondary spermatocytes are cylindrical cells with an apical nuclei, smaller but more elongated cell than the primary ones (Plate 1B).

Early-stage spermatids are small cylindrical with rounded nucleus and smaller than secondary spermatocyte. Middle-stage spermatid became more elongated with kidney shape nucleus and attached to Sertoli cells (Plate 1A). Late-stage spermatids are more differentiated with crescent shaped nucleus (Plate 1 A & B). Mature spermatozoa concentrated at the center of the male acini. They are much more elongated with fusiform-shaped head, longer mid-piece and tail (Plate 1A).

On the other hand, the female acini are characterized by the presence of oocytes associated with the basal layer of follicular cells in contact with the acinar epithelium. Oocytes were developed through successive previtellogenic and vitellogenic stages. Previtellogenic oocytes are small in size and have a basophilic cytoplasm (Plate 1C). Previtellogenic oocytes (I) or oogonia are the smallest cells with large nuclei surrounded with a thin layer of cytoplasm and have no follicular cells (Plate 1D). Previtellogenic Oocytes (II) are much larger than oogonia, with basophilic cytoplasm and large lightly stained nuclei (Plate 1C). Vitellogenic Oocytes (I) are large rounded cells with central rounded eosinophilic nuclei and some small follicular cells (Plate 1D). Vitellogenic Oocytes (II) have huge nuclei with nucleoli and their granular cytoplasm contains large amounts of yolk (Plate 1C). Vitellogenic oocytes (III) or mature oocytes have the largest amounts of yolk, large nuclei and huge number of follicular cells (Plate 1C & D).

#### 3.5.2 Histopathological alterations of hermaphrodite gland of snails treated with 2% Eucalyptus oil

**Plate 2:**
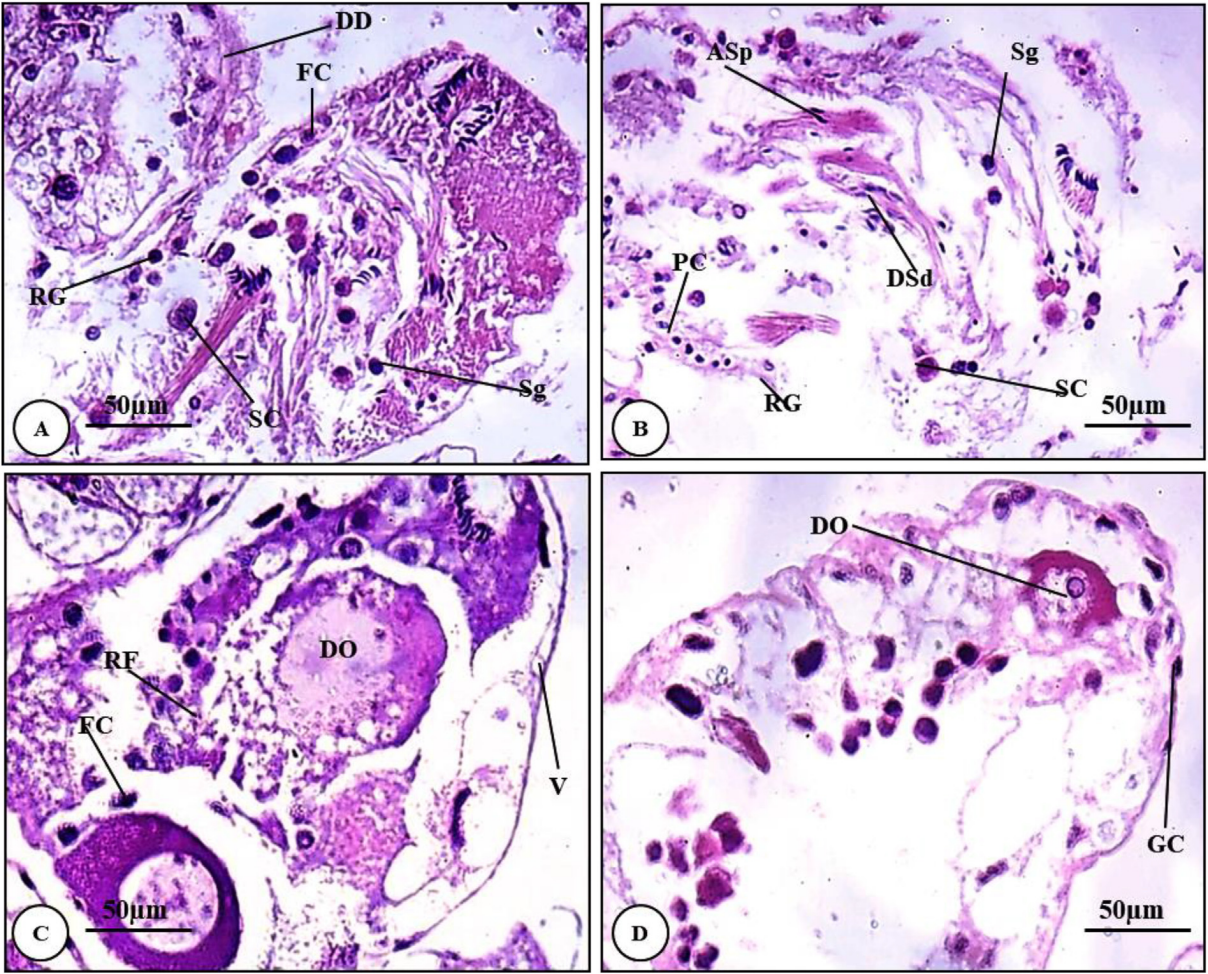
T.S. of hermaphrodite gland of *T. pisana* (Stained H&E) treated with 2% *Eucalyptus* oil (X = 400). (A & B) indicate deformation in stages of spermatogenesis. (C & D) show deteriorations in the follicular layer and oocytes (X = 400). *ASP: Adhered spermatozoa; DD: Deformed Digestive tubule; DO: Deteriorated oocyte; DSd: Deformed spermatid; FC: Follicular cell; PC: Picnotic germinal cell; RF: Ruptured Follicular layer; RG: Ruptured germinal layer; SC: Spermatocytes; Sg: Spermatogonia; V: Vacuole.*

The hermaphrodite gland of snails treated with 2% *Eucalyptus* oil suffered from disruption of hermaphrodite acini, ruptured and nuclear piknosis of germinal epithelium. Different stages of spermatogenesis including spermatogonia, spermatocytes, spermatids and even mature sperms were disrupted, decreased in number and almost diminished. Spermatids were disappeared and numbers of spermatozoa were reduced greatly. The spermatogenic cells in most of the acini were inhibited to develop into adult sperms (Plate 2A & B).

On the other hand, the germinal epithelial layer of female acini was destructed and vacuolated. Different stages of oogenesis were reduced and the mature oocytes became deformed (Plate 2C & D).

#### 3.5.3 Histopathological alterations of hermaphrodite gland snails treated with 2% Ricinus seeds extract

**Plate 3:**
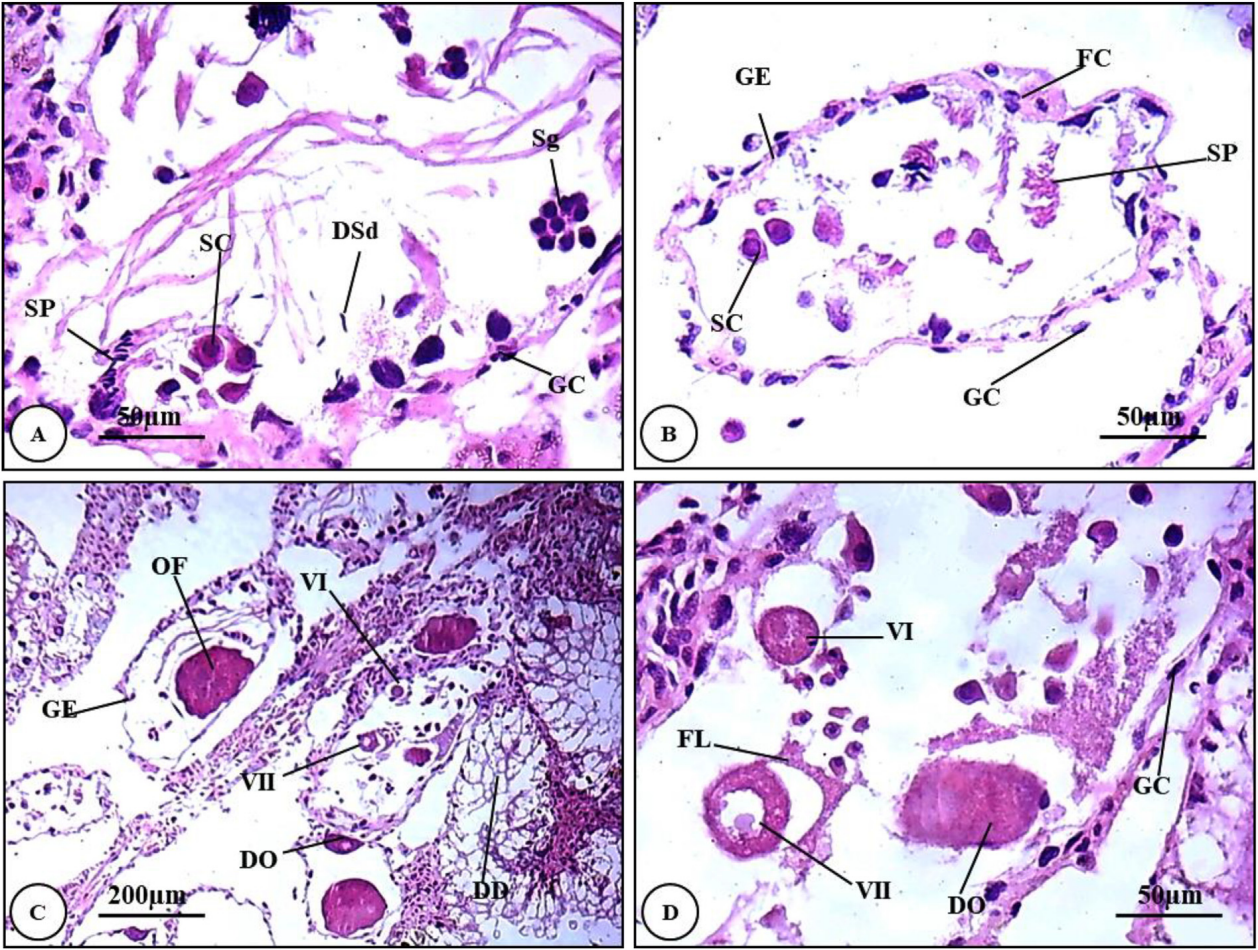
T.S. of the hermaphrodite gland of *Theba pisana* (stained H&E) treated with 2% methanolic extract of *Ricinus* seed showing disruption in acini. (A & B) show alterations in sperms and their stages (X = 400). (C) General view of hermaphrodite acini showing deformations in oocytes and their stages (X = 100). (D) T.S of the gland shows deteriorations in oogenesis stages (X = 400). *DD: Damaged digestive tubules; DO: Deformed oocyte; DSd: Deformed spermatid; FC: Follicular Cell; FL: Follicular Layer; GC: Germinal cell; GE: Germinal epithelial OF: Ovulated follicle; SC: Spermatocyte; Sg: Spermatogonia; Sp: Spermatozoa; VI: Vitellogenic oocyte I; VII: Vitellogenic oocyte II*.

The hermaphrodite acini of snails exposed to 2% methanolic extract of *Ricinus* seed was dramatically deteriorated. The germinal epithelial layer was ruptured, spermatogonia greatly decreased in number and their differentiation into spermatocytes was nearly inhibited. Spermatocytes were scanty and their chromatin material became condensed. Spermatids were deformed. Mature sperms were diminished (Plate 3A & B).

Different developmental stages of oogenesis showed severe distortions. Vitillogenic oocytes were disturbed. The germinal epithelium and follicular cells were ruptured. Mature ova were highly deteriorated (Plate 3C & D).

## 4 Discussion

Results of the present study demonstrated obvious effect of both used plant extracts on the reproductive biology of the land snail *Theba pisana*. *Ricinus* extract was found to possess more potent antifertility effects than *Eucalyptus* oil against *the* snails after 6 weeks of treatments. The highest concentration (2%) of *Ricinus* extracts caused complete ceasing of egg lying at the 6^th^ week while 2% *Eucalyptus* oil caused complete egg ceasing at the 5^th^ week of exposure. The effect of *Ricinus* extract may be due to the presence of phytosterols and alkaloids which resembles the effect of combined estrogen and progesterone in blocking the release of LH and FSH) that lead to the antifertility effect (Jehan et al., 1973; Sani and Sule 2007). According to Lamb et al. (1985), the toxic effect of the glycoprotein ricin in *Ricinus* extract may interfere with the enzymatic pathways of the reproductive system**.** Moreover, the methanolic extract of *Ricinus* was found to possess anti-ovulatory activity and distorted the estrous cycle of adult rats. Furthermore, ricin polypeptide chains A has a potent abortifacient effect on mice (Salhab, 1996; Sandhyakumary et al., 2003). *Ricinus* seed extract has also an injurious effect on male reproductive performance in rats and rabbits (Nazar, 2019; Raji et al., 2006) and exerted potential male contraceptive agents inhibiting spermatogenesis and motility of sperms (McNeil et al., 2021). On the other hand, *Eucalyptus* oil has high effect but somewhat lower than *Ricinus* extract on reproductive biology of the snail *T. pisana*. Similarily, *Eucalyptus* oil was found to reduce fecundity, egg hatchability, increased neonates larval mortalities and adverted offspring emergences of the beetles *Acanthoscelides obtectus* (Papachristos and Stamopoulos, 2002). The inhibitory effect of *Eucalyptus* oil on the reproductive biology of *T. pisana* snail may be due to the presence of its major component 1, 8-cineole (Hategekimana and Erler, 2020) in addition to other phenolic, alkaloid or saponin components (Lamirande and Gagnon, 1992, Singh et al., 2015).

The biological response of an organism to xenobiotics is induced by toxicants at the cellular and biochemical levels resulting in changes in physiology and behavior of the organism (Parvez and Raisuddin, 2005). Oxidative stress occurred after an imbalance in the biological oxidant-to antioxidant ratio producing oxidative damage to lipid, proteins, carbohydrates and nucleic acids (El-Demerdash, 2007). Antioxidant enzymes form defense line against oxidative stress (Ojha et al., 2011). The present study declared that 2% *Ricinus* extract caused highly significant decrease in the activities of CAT, SOD, GST and MDA enzymes. CAT and SOD are concentrated inside peroxisomes and are responsible for the degradation of hydrogen peroxide (Halliwell and Gutteridge, 1999). This depletion may be due to the excessive utilization of antioxidants to attenuate the free radicals generated as a result of the oxidative stress induced by the treatment of *Ricinus* extract (Parker et al., 2016). Contrarily, treatment with 2% of the *Eucalyptus* oil in this study resulted in elevations of CAT and SOD levels and significantly decreased GST and MDA levels. Elevation of both antioxidants as a result of cellular damage to inhibit the accumulation of reactive oxygen (ROS) intermediates (Radwan et al., 2010). These results are in line with Ugokwe et al. (2020) who reported significant increase in the activities of SOD and CAT in the digestive gland of *L. aurora* exposed to waste leachate. The toxic effect of *Eucalyptus* oil may be due to the presence of saponins (Singh et al., 2015), alkaloids (Khaki et al., 2011), flavonoids and/or phenolic compounds (Lamirande and Gagnon, 1992). GST is involved in catalyzing the conjugation of electrophilic substrates that protect cells from xenobiotic (Ferrari et al., 2007). MDA concentrations indicate the rate of lipid peroxidation within the organism (Liu et al., 2013). Severe decreases of antioxidant enzymes may be due to damages of the digestive gland which represent the main site of antioxidant formation (Zarai et al., 2011).

The current study showed that levels of LH and FSH decreased significantly upon exposure to the tested plant extracts. Such decrease may be due to inhibitory effects of *Eucalyptus* and *Ricinus* seeds extract on the secretion of gonadotropin releasing hormones. The extract of *Ricinus* seeds was reported to have anticonceptive, anti-ovulatory; inhibition of follicle development, anti-implantation, abortifacient effects due to the presence of alkaloids, flavonoids and polyphenols that affect steroidogenesis (McNeil et al. 2003; Raji et al., 2006; Meena and Roa, 2010; Hasegawa et al., 2013; Uadkla et al., 2013).

Thyroid-stimulating hormone stimulates thyroid hormones that promote carbohydrates; lipids and protein metabolism-the way the body use energy- and affect nearly all body organs (Magner, 2014; Delitala et al., 2019). Elevation of thyroid hormones is called hyperthyroidism and can cause speeding up of many body's functions and vice versa. It plays an important role during pregnancy both in the development of healthy baby and maintaining health of mother. Thyroid dysfunction (Hyperthyroidism or hypothyroidism) of TSH hormone increases the abortion chance in addition to fetal deformities (Alemu et al., 2016). Results in this study reported significant increase of Estrogen levels and decreased testosterone levels after treatments that may produce a decline in the sperm performance in snails. Similarly, *Ricinus* extract has a potential to be developed into a male contraceptive agent after reducing testosterone secretion (Raji et al., 2006). Ricinoleic acid derived from castor used as contraceptive jelly in folk medicine (Schulster et al., 2016). In this concern, testosterone levels in *Biomphalaria alexandrina* snails were decreased after exposure to *Haplophyllum tuberculatum* extract (Rizk et al., 2012), curcumin, *Callistemon citrinus* and *Zingiber officinale* extracts (El-Emam et al., 2017)and herbicides (Ibrahim and Sayed 2019). Prolactin is a hormone that stimulates the mammary gland to secrete milk and its high level decreases sex hormones (Freeman et al., 2000)**.** In this study, the levels of prolactin significantly increased after treatment and this may explains the gonadal dysfunction and could be attributed to the oxidative stress of these products on the snail. These results are in agreement with Abd El-Atti et al. (2020) who indicated significant increase of prolactin level in *Eobania* snails after treatment with a contraceptive drug.

The histological structures of the hermaphrodite gland of untreated *T. pisana* snails reported in this study showed different stages of spermatogenesis and oogenesis in addition to highlighting the follicular and germinal layers with their cells. Similar structures were illustrated by Morad (2021) for hermaphrodite gland of *Eobania vermiculata* snails and Rodrigues et al. (2021) for that of *Biomophalaria alexandrina*. The present study revealed disordered developmental stages in hermaphrodite acini of treated snails; *Eucalyptus* oil caused decline in sperm numbers and their stages in addition to destruction of germ cells and reduction in oocytes. *Eucalyptus* effect may be due to the presence of Eucalyptol (1, 8-cineole) (Hategekimana and Erler, 2020). On the other hand, *Ricinus* extract resulted in severe destruction in sperms leading to nearly complete inhibition. Moreover it caused rupturing of follicular layer and deterioration in oocyte stages. This may be due to the presence of alkaloids, flavonoids and polyphenols in *Ricinus* extract that had a spermicidal effect inhibition of steroidogenesis (Makonnen et al., 1999). This study declared that the levels of LH and FSH decreased significantly and so they affected the testosterone secretion and in turn affected the development of testis and stages of spermatogenesis. *Ricinus* extract was more effective upon gametogenesis. Similarly, Raji et al., 2006 found that the methanolic extract of *Ricinus* induced a disruption in the seminiferous tubule and erosion in germinal epithelial cells in the testes of treated rats.

## 5 Conclusion

The present work revealed that both tested plant extract induced severe reproductive, enzymatic, hormonal and histological disturbances of *T. pisana* snails. Baits containing these natural products were found to be strongly effective, ecofriendly and simply applicable technique for control of this pest. Reduction of egg laying capacities and hatching rates of treated snails give a promising hope of using these plant extracts in IPM programs.

## Declarations

### Author contribution statement

Mahmoud M.A. Desouky, Professor: Conceived and designed the experiments; Analyzed and interpreted the data.

Mahmoud S. Abd El-Atti, Professor: Conceived and designed the experiments; Wrote the paper.

Ali A. Elsheakh, Professor: Contributed reagents, materials, analysis tools or data.

Wesam S. Elgohary, Ph D student: Performed the experiments; Wrote the paper.

### Funding statement

This research did not receive any specific grant from funding agencies in the public, commercial, or not-for-profit sectors.

### Data availability statement

Data included in article/supp. material/referenced in article.

### Declaration of interest’s statement

The authors declare no competing interests.

### Additional information

No additional information is available for this paper.
